# Chronic Lymphocytic Leukemia: Molecular Pathologies and Therapeutic Strategies

**DOI:** 10.3390/ijms27115117

**Published:** 2026-06-05

**Authors:** Kelly Meza, Carla Barrientos Risso, Ankit Shah, Carla Romagnoli, Jose Sandoval, Yelida Brauchle, Alexandra Lyubimova, Leily Santos, Evelyn Goya Balaguer, Jacqueline Barrientos

**Affiliations:** 1 Department of Internal Medicine, Baylor College of Medicine, Houston, TX 77030, USA; kelly.meza-capcha@bcm.edu; 2 Baystate Medical Center, University of Massachusetts Chan Medical School, Springfield, MA 01107, USA; 3 Department of Medicine, Division of Hematology/Oncology, Rutgers New Jersey Medical School, Newark, NJ 07103, USA; 4 Rutgers Cancer Institute, Newark, NJ 07103, USA; 5 Mount Sinai Medical Center, Miami Beach, FL 33140, USA; 6 Moffitt Malignant Hematology & Cellular Therapy at Memorial Healthcare System, Pembroke Pines, FL 33028, USA

**Keywords:** chronic lymphocytic leukemia, BTK inhibitors, BCL-2 inhibitors, prognostic scores, TP53 abnormalities, IGHV, complex karyotype

## Abstract

Therapy for chronic lymphocytic leukemia (CLL) has evolved dramatically with the introduction of targeted agents, particularly Bruton tyrosine kinase inhibitors (BTKis) and BCL2 inhibitors (BCL2is). This review summarizes contemporary frontline and relapsed/refractory treatment strategies, with an emphasis on molecular risk stratification, combination and triplet regimens, measurable residual disease (MRD)–guided therapy, and time-limited approaches. We further examine how genomic complexity, prior therapies, and sociodemographic factors influence disease progression, treatment resistance, and clinical outcomes.

## 1. Introduction

Chronic lymphocytic leukemia (CLL) is a heterogeneous B-cell malignancy characterized by the clonal expansion of mature lymphocytes and wide variability in clinical behavior. Over the past decade, the therapeutic paradigm has shifted from chemoimmunotherapy (CIT) to targeted, mechanism-driven approaches, with Bruton tyrosine kinase inhibitors (BTKis) and BCL2 inhibitors (BCL2is) establishing chemotherapy-free regimens that improve progression-free survival (PFS) and tolerability, particularly in patients with high-risk genomic features or advanced age [1,2,3,4,5,6,7,8]. Molecular profiling, including cytogenetics, next-generation sequencing, and measurable residual disease (MRD) assessment, has refined risk stratification and treatment selection, and has informed the development of combination, triplet, and time-limited strategies aimed at maximizing depth of response while minimizing toxicity [8,9,10,11,12,13]. Genomic aberrations such as TP53 mutations, complex karyotype, and unmutated IGHV status, as well as prior therapy and demographic factors, have a profound impact on therapeutic outcomes and resistance patterns [1,2,3,4,5,6,7,8,9].

This review synthesizes contemporary frontline and relapsed/refractory CLL treatment strategies, emphasizing molecular pathogenesis, MRD-guided approaches, and evolving sequencing paradigms that integrate genomic and clinical insights to optimize patient care.

## 2. Evolving Therapeutic Landscape

### 2.1. Frontline Treatment Landscape

CLL has undergone a remarkable transformation in its therapeutic landscape. Historically managed with CIT regimens such as fludarabine, cyclophosphamide, and rituximab (FCR), frontline treatment strategies for CLL have progressively evolved toward more precise, targeted approaches. This shift has been driven by an improved understanding of the disease’s molecular pathogenesis and the development of novel agents that disrupt key survival pathways in CLL cells [10,11]. Two critical pathogenic pathways in CLL involve B-cell receptor (BCR) signaling–driven proliferation and resistance to apoptosis mediated by BCL2 overexpression. These mechanisms contribute to the accumulation of CLL cells, tissue infiltration, and immune dysregulation. BTK, a crucial mediator of BCR signaling, has emerged as a therapeutic target; BTK inhibitors (BTKis) offer chemotherapy-free management of B-cell malignancies and have not only improved PFS but also redefined long-term disease control, particularly in older patients and those with high-risk genetic features [12,13,14].

#### 2.1.1. BTK Inhibitors in the Front-Line Setting

BTK has shown clinical benefit in delaying disease onset and progression. Ibrutinib, the first-in-class irreversible BTKi, has demonstrated the ability to disrupt CLL cell signaling, adhesion, proliferation, and homing [13,14].

The RESONATE-2 trial, a pivotal phase 3 randomized study, evaluated ibrutinib in treatment-naïve patients with CLL aged 65 years or older without del(17p). At the 8-year follow-up, ibrutinib demonstrated a significant PFS benefit compared to chlorambucil, leading to an 85% reduction in the risk of disease progression or death, with durable responses even in high-risk subgroups, including advanced stages, bulky disease, and high-risk genomic features such as unmutated IGHV, del(11q), or TP53 mutations. At 7 years, PFS rates for ibrutinib-treated patients ranged from 52% to 68%, depending on genetic features, compared to substantially lower rates with chlorambucil. Median overall survival (OS) was not reached for Ibrutinib-randomized patients, but the 7-year survival estimate stood at 78%. The overall response rate (ORR) for patients assigned to ibrutinib was 92%, compared to 37% for those assigned to chlorambucil. Furthermore, responses to ibrutinib were durable, with the median duration of response not reached, while the median duration for chlorambucil treatment was 29.7 months. Adverse events (AEs) such as diarrhea, fatigue, cough, hypertension, and atrial fibrillation were common, though many improved with dose adjustments, and real-world evidence suggests that dose management can mitigate toxicity without compromising outcomes [15].

The ELEVATE-TN trial assessed acalabrutinib, a more selective second-generation covalent BTK inhibitor (cBTKi), in combination with obinutuzumab or as monotherapy, versus obinutuzumab plus chlorambucil in patients with previously untreated CLL. With a median follow-up of 28.3 months, both acalabrutinib-containing arms demonstrated significant PFS benefits [16]. In the 4-year follow-up of the study, the median PFS was not reached in the acalabrutinib-containing arms versus 27.8 months for the obinutuzumab-chlorambucil group. The estimated PFS at 24 months was 93% for the acalabrutinib-obinutuzumab group, 87% for acalabrutinib monotherapy, and 47% for obinutuzumab-chlorambucil. At 48 months, these rates were 87%, 77.9%, and 25.1%, respectively. The ORR was significantly higher with acalabrutinib-obinutuzumab combination [17]. These results were consistent with those seen in the iLLUMINATE trial, which evaluated ibrutinib with obinutuzumab versus chlorambucil plus obinutuzumab. Ibrutinib plus obinutuzumab reduced the risk of progression or death by 75% and decreased the need for next-line therapy by 96%, with an estimated 42-month PFS of 74% versus 33% in the comparator arm; high-risk subgroups also derived PFS benefit [18].

Zanubrutinib, another next-generation covalent, highly selective irreversible BTKi, has demonstrated potent BTK inhibition [19]. In the SEQUOIA phase 3 trial comparing zanubrutinib to bendamustine-rituximab in treatment-naïve patients without del(17p), zanubrutinib achieved superior 24-month PFS (85.5% vs. 69.5%) with consistent efficacy across subgroups, including Binet stage C, unmutated IGHV, and bulky disease. ORR was higher with zanubrutinib, and the median duration of response was not reached versus 30.6 months with bendamustine-rituximab. The estimated 24-month OS was similar (94.3% vs. 94.6%). In a non-randomized cohort with del(17p), zanubrutinib showed 24-month PFS of 88.9% and ORR of 90%. Zanubrutinib was well tolerated, with lower rates of cardiac arrhythmias and discontinuations due to AEs compared to the chemotherapy arm [20].

Together, these pivotal trials highlight significant advancements with BTKis in frontline CLL. While ibrutinib remains effective, its cardiovascular toxicity profile has steered practice toward more selective agents. Acalabrutinib and zanubrutinib have shown comparable or superior efficacy with improved safety; zanubrutinib offers a favorable balance of efficacy and tolerability, including in high-risk populations such as del(17p). The choice of BTKi in clinical practice should consider individual patient characteristics, comorbidities, and risk profiles, with next-generation agents increasingly preferred due to their safety advantages [20,21].

#### 2.1.2. BCL2 Inhibition and Time-Limited Therapy

One of the most significant advances in the frontline treatment of CLL has been the introduction of fixed-therapy, chemotherapy-free regimens that achieve deep and durable remissions. The landmark CLL14 trial evaluated such an approach, investigating the efficacy of fixed-duration therapy with venetoclax, a selective BCL2i, in combination with obinutuzumab in previously untreated patients with CLL and coexisting medical conditions. A total of 432 patients were randomized to receive either venetoclax plus obinutuzumab or chlorambucil plus obinutuzumab. The study population included individuals with high-risk features such as unmutated IGHV, del(17p), and/or TP53 mutations [21]. A key advantage of the venetoclax plus obinutuzumab regimen was its fixed-duration administration, which produced durable responses that extended well beyond the end of therapy. Patients receiving this regimen experienced a significantly longer 5-year PFS compared with those receiving chlorambucil plus obinutuzumab (5-year PFS 62.6% vs. 27%). This benefit was maintained among high-risk subgroups, including patients with del(17p) and/or TP53 mutations (5-year PFS 40.6% vs. 15.6%) and those with unmutated IGHV (5-year PFS 55.8% vs. 12.5%) [21].

Time to next anti-leukemic treatment (TTNT) was also prolonged in the venetoclax plus obinutuzumab arm, with 72.1% of patients remaining treatment-free at 5 years, compared with 42.8% in the chlorambucil plus obinutuzumab group. Although patients with high-risk genomic alterations had shorter overall TTNT, those receiving venetoclax plus obinutuzumab clearly benefited from the time-limited approach. Importantly, achieving undetectable MRD at the end of therapy was associated with longer PFS, and this deep response correlated with improved OS. While 5-year OS did not significantly differ between arms (81.9% for venetoclax plus obinutuzumab vs. 77% for chlorambucil plus obinutuzumab), patients with del(17p) and/or TP53 mutations experienced poorer outcomes in both groups, emphasizing the need for individualized treatment strategies in high-risk populations [22].

#### 2.1.3. Combination Therapies and Chemo-Free Regimens

Despite previous successes in drug development, the ongoing need for continuous treatment, the low rates of achieving complete response (CR), and the inability to reach undetectable MRD (uMRD) have highlighted the necessity for alternative treatment approaches. To address this, the CLL13/GAIA study was conducted for treatment-naïve patients. In this study, participants were assigned to one of four groups: one group received CIT, the second group a combination of venetoclax and rituximab, the third group received a combination of venetoclax and obinutuzumab, and the fourth group received a combination of venetoclax, obinutuzumab, and ibrutinib. 4-year PFS rates were 85.5% for the triple combination group, 81.8% for the venetoclax-obinutuzumab group, 70.1% for the venetoclax-rituximab group, and 62% for the CIT group. The high PFS rates observed in the first two groups are comparable to those seen with continuous BTKi therapy in similarly young and fit patient cohorts, such as in the FLAIR trial [23]. OS did not significantly differ across groups. However, patients with unmutated IGHV appeared to benefit from the MRD-guided triple combination compared to those treated with the doublet of venetoclax and obinutuzumab. In contrast, no benefit was observed for the subgroup with mutated IGHV or across the overall population. This study found that MRD status in peripheral blood at month 15 correlated with PFS, and second-line treatments mostly consisted of BTK inhibitor-based and venetoclax-based therapies. However, the triplet regimen was associated with higher rates of severe infections and cardiac events, and no OS advantage was seen among venetoclax-based groups. Limitations included exclusion of TP53 aberrant cases and a relatively short observation period with few PFS/OS events [24].

When considered together, MRD-guided strategies, such as those in the triplet arm of GAIA/CLL13 and the FLAIR trial, have the potential to extend PFS compared with uniform fixed-duration approaches using similar combinations. The FLAIR trial, a phase 3 multicenter study, initially randomized fit patients with previously untreated CLL to receive either ibrutinib plus rituximab or FCR. In 2017, the trial was adapted to include ibrutinib monotherapy and ibrutinib plus venetoclax, with treatment duration guided by MRD status [24,25]. In contrast, the GLOW trial, an international phase 3 study, enrolled older or less fit patients and compared ibrutinib plus venetoclax to chlorambucil plus obinutuzumab, but used a fixed 12-cycle duration regardless of MRD status [26]. Despite these differences, both trials demonstrated superior PFS and deeper responses with ibrutinib plus venetoclax. In FLAIR, 3-year PFS was 97.2% versus 76.8% with FCR, and 3-year OS was 98.0% versus 93.0%. By 5 years, 65.9% of patients had uMRD in bone marrow and 92.7% in peripheral blood, with MRD results guiding early therapy discontinuation in responders. In GLOW, median PFS was 38.7 versus 21.0 months with chlorambucil plus obinutuzumab, and the rate of uMRD in peripheral blood three months after completing therapy was 54.7% for ibrutinib plus venetoclax versus 17.1% in the comparator group, demonstrating that even a fixed-duration regimen can achieve durable depth of response [27].

Both trials also evaluated the safety profile of ibrutinib plus venetoclax, revealing manageable yet notable AEs. In both studies, the most common grade ≥ 3 AE was neutropenia, with infections being the most frequent AE in the FLAIR study. Regarding cardiac events, atrial fibrillation and hypertension were among the most frequent AEs. The rate of treatment discontinuation due to AEs was similar in both studies, at around 20% in FLAIR and 19.1% in GLOW. Tumor lysis syndrome was rare in both trials [24,26]. Overall, both trials underscore the potential of ibrutinib plus venetoclax as a time-limited, chemotherapy-free regimen for the first-line treatment of CLL, offering deep and durable remissions. The FLAIR trial supports the benefit of MRD-guided treatment duration, which may help minimize therapy exposure for responders. Meanwhile, the GLOW trial confirms that even a fixed 12-cycle regimen can significantly improve PFS and uMRD rates in a less fit population compared to conventional CIT [24,26]. Based on this, the combination of I + V was approved in Europe and Canada [28,29,30,31].

### 2.2. Doublet and Triplet Regimens in Development

In CLL, doublet regimens typically combine a BCL2i, such as venetoclax, with an anti-CD20 monoclonal antibody, such as obinutuzumab or rituximab, whereas triplet regimens add a BTKi, such as ibrutinib, acalabrutinib, zanubrutinib, or pirtobrutinib, to this backbone. The rationale for triplet therapy is to achieve deeper responses, including higher rates of (uMRD), extend PFS and OS, and enable longer treatment-free intervals in fixed-duration regimens [32,33].

The CLL13 trial investigated the regimen venetoclax-obinutuzumab-ibrutinib for 12 cycles as compared to 12 cycles of venetoclax-obinutuzumab or venetoclax-rituximab or 6 cycles of CIT (bendamustine-rituximab or fludarabine-cyclophosphamide-rituximab) in fit patients with low burden of co-existing conditions without TP53 mutation or 17p deletion. In this trial, ibrutinib was discontinued upon 2 consecutive MRD-negative measurements. The trial found that at 15 months, patients treated with the triplet regimen had a 92.2% rate of uMRD, which was superior to CIT (52%). 3-year PFS was 90.5% in this triplet arm, which was superior to CIT (HR: 0.42). This study was not designed to compare the triplet regimen to the venetoclax-obinutuzumab doublet. While venetoclax-obinutuzumab-ibrutinib was superior to CIT in the entire study population, this difference appeared limited to the IGHV unmutated patients on subgroup analysis. MRD negativity notably correlated with prolonged PFS. Out of 198 patients who completed the trial on the triplet regimen, 177 were able to successfully discontinue treatment, suggesting the prospect of a treatment-free interval [34].

More recently, the AMPLIFY study evaluated fixed-duration acalabrutinib-venetoclax with or without obinutuzumab vs. CIT (FCR or BR) in CLL patients without TP53 mutation or 17p deletion. In this trial, acalabrutinib-venetoclax resulted in 83.1% 3-year PFS, which was the highest out of the 3 treatment arms. The triplet combination resulted in an MRD negativity rate of 66.4%. It is worth noting that OS was worse with the triplet regimen compared to venetoclax-acalabrutinib doublet, and this difference was attributed to infectious complications during the COVID-19 pandemic. This highlights that the addition of obinutuzumab may result in deeper remission and a longer PFS but at the cost of immunosuppression [35]. These positive findings led to the recent FDA approval of the combination of acalabrutinib and venetoclax, marking the first all-oral, fixed-duration regimen available in the frontline setting.

In the phase 2 CLL2-BZAG trial, the triple combination of zanubrutinib, venetoclax and obinutuzumab was studied after optional bendamustine debulking in patients with relapsed/refractory CLL. In this cohort of 42 patients, the median number of prior lines of therapy was 1 and 15 (37.5%) had TP53 mutation, and 31 (77.5%) had unmutated IGHV. In the second line setting, the triplet combination resulted in a 52.5% uMRD rate at 6 months and a best uMRD rate of 85% with prolonged therapy. PFS and OS at 18 months were 96% and 96.8%, respectively, with COVID-19 infection as the main AE. The triplet is also being evaluated in first-line CLL/SLL and mantle cell lymphoma, with early mantle cell data showing uMRD rates of 95% at 10^−5^ and 84% at 10^−6^ [23,36].

A recent publication by Al-Sawaf et al. reported the first analysis of the phase III CLL17 trial. In this study, patients with previously untreated CLL were randomized to receive fixed-duration therapy with venetoclax-obinutuzumab or venetoclax-ibrutinib, or continuous ibrutinib monotherapy. The results demonstrated that fixed-duration targeted therapy with venetoclax–obinutuzumab or venetoclax–ibrutinib was noninferior to continuous ibrutinib. The absence of superiority for continuous ibrutinib in this study challenges the historical assumption that indefinite BTK inhibition is required to maintain disease control. Notably, patients with unmutated IGHV did not experience inferior outcomes with fixed-duration therapy compared with continuous ibrutinib, supporting the feasibility of time-limited treatment strategies. Among patients with del(17p) and/or TP53 mutation, outcomes appeared favorable with BTKi–containing regimens. However, the small size of this subgroup (<8%) and the limited follow-up duration preclude definitive conclusions. Importantly, continuous ibrutinib did not demonstrate superiority over fixed-duration venetoclax–ibrutinib [37].

Lastly, the triplet regimen of pirtobrutinib, venetoclax and obinutuzumab is under study in untreated CLL (NCT05536349). The trial cohort included 74 patients, of which 77% had unmutated IGHV and 12% had del(17p)/TP53 mutation. After 7 cycles of therapy, this treatment resulted in a uMRD6 (<10^−6^) rate of 65% in the marrow and 79% in the blood. uMRD4 rates (<10^−4^) after C7 were 91% in bone marrow and 93% in blood. At the end of 13 cycles, the uMRD6 rates improved to 81% in marrow and 89% in blood with respective uMRD4 rates of 96% (bone marrow) and 100% (blood). Importantly, no patients progressed, died or, to our best knowledge, discontinued due to adverse effects. As a follow-up for this trial, short-term and more patients have yet to reach the C7 and C13 time-points. Nonetheless, the preliminary data are highly encouraging that this triplet regimen can result in very deep remissions with good tolerability [1].

Taken together, doublets have established proof-of-concept for fixed-duration regimens and chemotherapy-free therapy, while triplet regimens have demonstrated the potential to achieve deeper remissions, higher uMRD rates, and prolonged PFS, making them attractive options for first-line therapy in select patients [1].

### 2.3. Treatment Sequencing After Resistance for Clinical Practice

Following failure of BTKi, subsequent treatment options include venetoclax-based therapy with or without an anti-CD20 monoclonal antibody, or a non-covalent BTKi such as pirtobrutinib. Selection among these options depends on patient fitness, prior treatment tolerance, mutational profile, and depth and duration of response to prior therapy. In patients with disease progression after exposure to both BTK and BCL2i, therapeutic strategies include CD19-directed CAR T-cell therapy (e.g., lisocabtagene maraleucel, with or without concurrent BTKi), investigational BTK degraders, or allogeneic stem cell transplantation in carefully selected high-risk patients [38,39].

Clinical trial data support the activity of non-covalent BTK inhibition in this setting. The BRUIN CLL-321 study demonstrated that pirtobrutinib retains clinically meaningful efficacy in patients previously exposed to both cBTKi and venetoclax, including those harboring diverse BTK resistance mutations. Nevertheless, cumulative toxicity, cardiovascular AEs, and the development of resistance remain important concerns, particularly among patients with TP53 aberrations and unmutated IGHV [40,41].

In clinical practice, second-line therapy is largely guided by the nature of frontline treatment. Patients initially treated with a cBTKi are typically transitioned to venetoclax-based regimens at relapse, whereas those who received venetoclax-based frontline therapy are generally directed toward cBTKi-based treatment.

Resistance to cBTKis is most commonly mediated by mutations at the BTK C481 residue, which impair irreversible drug binding and reduce therapeutic efficacy. Data from the MURANO and CLL14 trials support venetoclax in combination with an anti-CD20 monoclonal antibody as an effective strategy in the relapsed/refractory setting [32,33]. Furthermore, findings from the CLL11 and CLL13 trials support venetoclax plus obinutuzumab as a preferred second-line regimen following cBTKi-based frontline therapy [22].

Given that CLL subclones may retain partial sensitivity during treatment transitions, overlapping therapy between cBTKi and venetoclax is often recommended, even when an anti-CD20 monoclonal antibody is included [42]. In patients who relapse following venetoclax-based therapy, re-treatment with venetoclax may be feasible, particularly when initial discontinuation was planned rather than driven by resistance. Although prospective data are limited, the largest retrospective series evaluating venetoclax re-treatment reported a median interval of 16 months between first and subsequent venetoclax-based regimens. In this cohort, venetoclax was administered as monotherapy (45.7%) or in combination with rituximab (28.2%), obinutuzumab (10.9%), ibrutinib (4.4%), or other agents (10.9%), resulting in an ORR of 80% and a median PFS of 25 months [21,43].

Progression after intolerance or after completion of fixed-duration treatment with BTKi and venetoclax should be approached differently than progression occurring while on continuous therapy. Several ongoing studies are specifically evaluating retreatment strategies after prior exposure to both BTK and BCL2 inhibition. The phase 2 MAVRiC trial (NCT07024706) is the first prospective study designed to evaluate mutation-guided finite-duration retreatment with acalabrutinib plus venetoclax in patients with relapsed CLL/SLL after frontline fixed-duration cBTKi plus BCL2 inhibitor–based therapy. The study stratifies treatment duration according to molecular risk features, including IGHV mutation status and TP53 aberrations, with the goal of determining whether biologic risk-adapted retreatment can prolong remission duration [44]. Similarly, the ReVenG trial (NCT04895436), a phase 2 study evaluating retreatment with venetoclax plus obinutuzumab (VenG) in patients previously exposed to the same regimen, demonstrated promising preliminary efficacy, with 100% of evaluable patients achieving a response upon retreatment [45,46]. In the MURANO study, 25 patients who relapsed after venetoclax–rituximab (VenR) were retreated with the same regimen, achieving an ORR of 72% and a median PFS of 23 months. The median interval from the last venetoclax dose to retreatment was 2.3 years. Notably, prolonged exposure to venetoclax beyond 2 years may facilitate the emergence of resistance-associated mutations; however, the clinical significance of these variants remains uncertain, as they did not appear to substantially impair responses to venetoclax retreatment [47].

Upon further disease progression or treatment intolerance, third-line options converge for both pathways and include pirtobrutinib, lisocabtagene maraleucel, enrollment in clinical trials, and emerging targeted agents [21,43,48]. Pirtobrutinib, a highly selective, non-covalent BTKi, represents a key therapeutic advance for patients progressing after cBTKi exposure. Unlike first- and second-generation BTKis, which irreversibly bind BTK at the C481 residue, pirtobrutinib reversibly inhibits both wild-type and C481-mutant BTK. Based on results from the phase 1–2 BRUIN trial, pirtobrutinib received FDA approval for patients with CLL previously treated with both a cBTKi and venetoclax. Off-label use may also be considered in patients who have progressed on venetoclax [49].

For patients with disease progression following both BTK and BCL2i therapy, additional therapeutic approaches include BTK degraders, CAR T-cell therapy, and allogeneic stem cell transplantation. BTK degraders are designed to overcome resistance mediated by BTK mutations by targeting the protein for proteasomal degradation. Agents currently under investigation include BGB-16673, NX-2127, and NX-5948, with early-phase data suggesting favorable tolerability and promising clinical activity [43,50].

#### CAR T-Cell Therapy

Lisocabtagene maraleucel (liso-cel) is an autologous CD19-directed CAR T-cell therapy developed by Bristol Myers Squibb and received FDA approval on 14 March 2024, for patients with relapsed or refractory CLL following prior treatment with both a BTKi and a BCL2i. This approval was based on results from the phase 1–2 TRANSCEND CLL 004 trial [4]. At dose level 2, among 87 treated patients, the independently assessed CR or CR with incomplete hematologic recovery (CRi) rate was 18% (95% CI, 11–28%) at a median follow-up of 21 months. The ORR was 47% (95% CI, 36–58%), with uMRD achieved in 64% of peripheral blood samples (95% CI, 53–74%) and 59% of bone marrow samples (95% CI, 48–69%). Best overall responses included CR/CRi (18%), partial or unconfirmed partial responses (29%), stable disease (39%), and progressive disease (7%) [9,43,50]. Furthermore, recent studies have combined liso-cel + ibrutinib, demonstrating substantial efficacy with deep remissions (86% ORR, 45% CR rate, and 86% blood uMRD rate) and manageable safety in pts with R/R CLL/SLL [51].

## 3. Molecular Pathogenesis and Prior Lines of Treatment

Prior lines of chemotherapy and immunotherapy have been shown to influence the development of high-risk mutations such as TP53 mutations, ATM deletions, and complex karyotypes. These genetic abnormalities are more frequently observed in relapsed/refractory patients and those exposed to fludarabine-based regimens, which may contribute to genomic instability [52].

Karyotype complexity, where we quantify the number of chromosome aberrations in a karyotype, has proven in many ways to be a molecular marker of prognosis. In CLL, complex karyotypes (CK), those with ≥3 chromosome abnormalities, are associated with poor outcomes in standard chemoimmunotherapy (CIT), and most recently have been shown to also have prognostic value in the setting of targeted therapies. We can further group patients into highly complex karyotypes (hCK), those with ≥5 chromosome abnormalities. In fact, high CK has consistently shown poor clinical outcomes regardless of disease stage, TP53 abnormalities, or whether they expressed mutated or unmutated immunoglobulin heavy variable genes. In contrast, cases with 3 or 4 chromosomal abnormalities only experienced aggressive disease progression when TP53 mutations were present [53].

Researchers analyzed data from the GAIA/CLL13 trial, including patients without TP53 mutations, and found that hCKTs and translocations are independent prognostic factors for shorter PFS. Standard CIT (FCR-BR) increased karyotypic complexity over time, but venetoclax-based treatments did not [53]. Patients with hCKTs had a significantly shorter PFS compared to those with CKTs (3 or 4 aberrations). While CKTs were associated with inferior outcomes in CIT, only hCKTs, rather than all CKTs, were independently predictive of worse prognosis in venetoclax-treated patients. Patients with hCKTs had a nearly two-fold increased risk of disease progression compared to those without hCKTs (HR: 1.96) [3]. This suggests that for CLL patients receiving venetoclax-based treatment, the extent of chromosomal complexity is a key factor influencing their therapeutic outcomes. In a retrospective study to elucidate the prognostic value of karyotype complexity in CLL patients treated with ibrutinib, patients with ≥5 cytogenetic abnormalities had significantly worse outcomes compared to those with fewer abnormalities. The hazard ratio for PFS was 1.07 (95% CI, 1.04–1.10; *p* < 0.0001), and for OS, 1.09 (95% CI, 1.05–1.12; *p* < 0.0001). The presence of karyotype evolution at disease progression, functioning as a marker of genomic instability, was also prognostic of subsequent survival, thus reinforcing the importance of sequential cytogenetic analysis [53].

### 3.1. Low Variant Allele Frequency TP53 Mutation

Next-generation sequencing has been useful in identifying CLL cells with low (0.1–10% and by other means undetectable) TP53 Variant Allele Frequency (VAF). Interestingly, when patients were treated with BTKi or other targeted therapies, low-VAF TP53 mutations did not negatively impact survival outcomes. This suggests that targeted therapies may mitigate the adverse effects of low-VAF TP53 mutations. However, in patients treated with CIT, low-VAF TP53 mutations may still have a negative impact on survival [4].

Analysis of the clonal evolution of low-VAF TP53 mutations showed that the highest expansion rates were associated with fludarabine, cyclophosphamide, and rituximab regimen in both first- and second-line treatments. In the relapsed patients, 33% had low-VAF TP53 mutations, which did not expand significantly upon targeted treatment. This suggests CIT could drive clonal evolution of TP53-mutated subclones. Furthermore, patients with TP53 mutations of 1–10% VAF and unmutated IGHV have worse survival outcomes, while those who switch to targeted therapies tend to benefit regardless of TP53 mutational status [4,5].

### 3.2. TP53 Mutations and Del(17p)

TP53 mutations and del(17p) represent well-established high-risk genomic abnormalities in CLL and are strongly associated with resistance to conventional therapies. Del17p is a high-risk genetic abnormality where there is a deletion of the short arm (p) of chromosome 17, which includes the *TP53* gene, a crucial tumor suppressor. Del(17p) and/or TP53 mutations on their own can result in the loss of wild-type p53 function. CLL patients with TP53 mutations have significantly shorter OS (median of 79.6 months) compared to those without mutations. Del(17p) is found in 5–10% of patients at diagnosis but in up to 40% of patients relapsing after fludarabine-based therapies [6,7,54]. Both TP53 mutations and del17p are independent predictors of shorter time to chemo-refractoriness. When a deletion of one TP53 allele (del17p) is combined with a mutation in the other allele (TP53m), it is called a double hit or biallelic inactivation. Using Sanger sequencing, data have shown that approximately 80% of patients harboring del(17p) also carry TP53 mutations in the second allele [7,8].

## 4. Genomic Landscape Stratified by Demographics

Recent sequencing data suggest differential expression and mutation frequencies of key CLL-related genes when stratified by age, race, and gender.

Age is a significant factor, as older patients are more likely to exhibit high-risk genomic features such as TP53 mutations, del(17p), and unmutated IGHV genes. These genetic changes are linked to resistance to CIT and poorer clinical outcomes. In contrast, younger patients exhibit a greater frequency of mutated IGHV status, typically experiencing a less severe disease progression, which makes them better candidates for watchful waiting or targeted therapies [55,56].

There are also noticeable differences in the biology of CLL based on sex. The disease more often impacts men and typically displays genomic changes related to unfavorable prognosis, such as mutations in NOTCH1, SF3B1, and complex karyotypes. These genetic alterations have been associated with a more aggressive disease course and increased risk of Richter’s transformation. Although female patients are less frequently affected, they may carry a different mutation profile and demonstrate better OS, possibly due to lower tumor burden and favorable IGHV mutation status [57,58].

Racial and ethnic variations in CLL have been the subject of growing interest, especially with the expansion of population-based genomic databases. Previous studies have demonstrated that African American patients present with higher frequencies of unfavorable prognostic markers such as unmutated IGHV status, higher ZAP 70 expression, del(17p) and 11q chromosomes, and TP53 and NOTCH mutations [59,60].

Hispanic and Asian populations exhibit lower incidences of CLL but have unique molecular profiles. For instance, Chinese patients with CLL show different frequencies of mutations in genes such as SF3B1, TP53, and NOTCH1 compared to patients of European descent. Specifically, the mutation frequencies in Chinese patients are 5% for SF3B1, 15% for TP53, and 8% for NOTCH1, which differ from those observed in European populations. Additionally, Taiwanese patients with CLL have a distinct immunoglobulin heavy chain variable region gene repertoire and a lower frequency of del(11q) compared to Western populations [61,62].

Disparities in access to advanced therapies may further compound differences in molecular evolution and outcomes. These differences are still brought on by variations in treatment accessibility and the level of treatment. Race, for example, significantly impacts the accessibility of contemporary therapies such as small-molecule inhibitors. Even though African American patients are increasingly using these treatments, their OS rates are still lower than those of White patients. These inequalities are worsened by socioeconomic factors as well, since individuals with a lower socioeconomic background tend to have worse CLL outcomes [63,64].

In conclusion, the genomic landscape of CLL varies significantly across different demographic groups, influencing disease presentation, progression, and outcomes.

## 5. Clinical and Molecular Predictors of Treatment Failure

### 5.1. Emergence of Resistance Mutations

#### 5.1.1. Resistance Mutations to Covalent BTK Inhibitors

**Figure 1:**
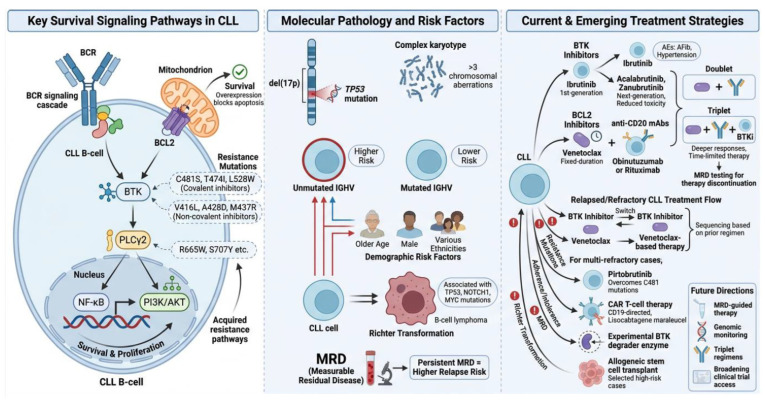
Key signaling pathways, molecular pathology, and treatment strategies in CLL. The figure summarizes major survival pathways involved in CLL pathogenesis, including BCR, BTK, PI3K/AKT, NF-κB, and BCL2 signaling; important molecular and prognostic factors such as TP53 alterations, IGHV status, complex karyotype, MRD, and Richter transformation; and current and emerging treatment approaches, including BTK inhibitors, venetoclax-based regimens, anti-CD20 antibodies, cellular therapies, and MRD-guided strategies.

Covalent BTKi targets the C481 residue of BTK, irreversibly binding to it and obstructing the ATP-binding site, thereby inhibiting the enzyme’s catalytic function. While these inhibitors have shown remarkable efficacy in treating CLL, many patients eventually develop resistance to the therapy [65] (Figure 1).

**C481S mutation:** The most prevalent mutation at the BTK C481 site is C481S, which modifies the conformation of the binding site, thereby reducing the ability of cBTKis to bind effectively. Consequently, BTK signaling can persist despite the presence of these inhibitors.

**PLCγ2 mutation:** Several mutations in PLCγ2 have been identified in cases of ibrutinib-resistant CLL, with most occurring in the SH2 domain, particularly mutations such as P664S, R665W, and S707Y. Importantly, these SH2 domain mutations have been shown to activate PLCγ2 independently of BTK, thereby restoring BCR signaling even in the presence of BTK inhibition. This mechanism likely explains their emergence under selective pressure from BTK inhibitor therapy.

**T474I mutation:** Like the C481S mutation, the T474I mutation enhances BTK autophosphorylation at Y223 even in the absence of BCR stimulation, thereby activating downstream signaling pathways. This mutation has been observed in relapsed/refractory (R/R) CLL patients treated with ibrutinib and acalabrutinib, but notably, it has not yet been reported in patients receiving zanubrutinib. This fact suggests that resistance mechanisms to cBTKis are not universally shared across all BTKis but may instead be specific to each individual inhibitor.

**L528W mutation:** Classified as a kinase-deficient or kinase-dead resistance mutation, has been reported in patients treated with both ibrutinib and zanubrutinib. This mutation is associated with diminished autophosphorylation at BTK-Y223 and a corresponding reduction in BTK enzymatic activity.

#### 5.1.2. Resistance to Non-Covalent BTK Inhibitors and BTK Degraders

Non-covalent BTKis were developed to improve on the pharmacologic properties of cBTK inhibitors while also maintaining potency against BTK C481 mutations. These drugs do not require binding to the BTK C481 residue and effectively inhibit wild-type and mutant BTK with C481 substitutions. Pirtobrutinib was identified to target BTK mutations and downstream PLCγ2 mutations, allowing escape from BTK inhibition. Specifically, a key set of BTK mutations was identified in 9 out of 55 patients with CLL refractory to pirtobrutinib, including V416L, A428D, M437R, T474I, and L528W. All these mutations are clustered in the kinase domain of BTK and can confer resistance to both covalent and noncovalent BTKi. Enzymatic analysis demonstrated that the V416L, A428D, M437R, and L528W resulted in diminished BTK enzymatic function, while the T474I resulted in enhanced BTK enzymatic function. Notably, the T474I and the L528W have also been described in the context of covalent BTK inhibition, highlighting the fact that common mechanisms and resistance can occur regardless of the covalent or non-covalent nature of the BTK inhibition. Besides the BTK mutations, genetic studies revealed that PLCγ2 mutations can also confer resistance to pirtobrutinib [66].

Based on the BRUIN trial, several factors were linked to disease progression within 24 cycles of pirtobrutinib in CLL patients. Those who had progressed on prior cBTKis were at higher risk of disease progression compared to patients who were intolerant, most of whom had wildtype BTK, highlighting the importance of BTK mutation status. Complex karyotypes were also associated with disease progression, with patients harboring TP53 mutations and/or del(17p), indicating a high-risk genetic profile. Baseline BTK mutations were more frequent in the disease progression group; although these patients initially responded, many developed new non-C481 BTK mutations, particularly those previously refractory to cBTKi. In contrast, patients with wild-type BTK/PLCG2 had longer remissions, suggesting better outcomes in BTKi-naive individuals. All three patients with mutated IGHV remained on therapy, consistent with prior findings of better prognosis in this subgroup. Notably, all five patients with XPO1 mutations experienced disease progression, suggesting a potential role in resistance that warrants further validation in the full BRUIN CLL cohort [67].

BTK protein degraders represent a novel class of therapeutics with a distinct mechanism of action compared to covalent and non-covalent BTK inhibitors. Instead of inhibiting BTK activity, these agents promote its ubiquitination, leading to proteasomal degradation. Promising preclinical studies suggest that this approach can effectively target and eliminate BTK, including forms with mutations that confer resistance to traditional BTK inhibitors [68].

### 5.2. Suboptimal MRD Clearance

Failure to achieve deep MRD negativity reflects incomplete eradication of leukemic clones and is increasingly recognized as a predictor of inferior long-term outcomes. Persistent MRD may arise from genetically complex subclones with intrinsic resistance, inadequate treatment duration or intensity, or sanctuary disease within protective microenvironments. In fixed-duration regimens, residual MRD is associated with earlier relapse following therapy discontinuation, whereas during continuous treatment, persistent MRD may facilitate clonal evolution and the subsequent emergence of resistance mutations [22,69].

### 5.3. Poor Adherence or Intolerance to Therapy

Suboptimal adherence, dose interruptions, or premature treatment discontinuation due to toxicity can compromise sustained pathway inhibition and contribute to treatment failure. AEs such as atrial fibrillation, bleeding, cytopenias, or infections frequently necessitate treatment modification, particularly with first-generation BTKis. Although intolerance-related discontinuation is biologically distinct from mutation-driven resistance, reduced drug exposure may permit residual disease persistence and clonal expansion, ultimately leading to disease progression [70,71].

### 5.4. Richter Transformation

Richter transformation is the evolution of CLL into an aggressive large B-cell lymphoma, representing a distinct and highly aggressive mechanism of treatment failure. It is more commonly observed in patients with high-risk molecular features, including TP53 mutations, prior exposure to chemoimmunotherapy, and genomic alterations involving NOTCH1 and MYC. Unlike gradual resistance in CLL, Richter transformation reflects clonal divergence and is associated with rapid clinical progression, poor response to standard CLL therapies, and inferior survival outcomes [64,65].

## 6. Complexities and Challenges of Managing CLL

CLL management has advanced with the commencement of targeted therapies. However, the management of CLL remains complex due to its variable clinical course. There are many challenges when dealing with patient comorbidities, therapy-related toxicities, uncertainties in therapeutic sequencing, and disparities in access to care [9].

Most patients are diagnosed at the age of 70 years, which is considered the median age of diagnosis [10]. Many patients present at the time of diagnosis with comorbidities such as chronic kidney disease and cardiovascular disease, increasing the risks of treatment-related adverse effects. Even when treating a patient with a targeted agent, they can be susceptible to toxicities. For example, prolonged use of BTKi is associated with increased risk of arrhythmias, hypertension, infection complications, and bleeding events [11,72,73,74]. Moreover, BTK inhibition is known to cause immunosuppression, raising concerns for fungal and viral reactivations. Therefore, patients on long-term BTKi should undergo regular cardiovascular monitoring and receive appropriate infection prophylaxis according to local guidelines [75].

Strategically selecting a therapy over the course of the disease, while aiming to maximize efficacy and minimize toxicities while preserving future treatment options, is referred to as therapeutic sequencing. CLL therapies include a wide range of agents, each with different mechanisms of action, efficacy, and safety profiles. Options for treatments, as discussed before, include continuous BTKi therapy or fixed-duration venetoclax plus obinutuzumab regimens. While having multiple treatment options is beneficial, it raises uncertainties about which treatment to use first, what combinations to apply, and what therapies to reserve for relapse [25]. Determining the optimal first-line therapy remains a challenge, each with distinct advantages and disadvantages. In the relapsed/refractory setting, sequencing of agents post-BTKi or BCL2i failure is not standardized, and clinical trial data are evolving. Novel combinations and next-generation agents, such as non-covalent BTKi like pirtobrutinib, offer promising salvage options but are not yet widely available [76].

Clinical trial enrollment for CLL disproportionately underrepresents elderly patients, racial minorities, and those from rural or low-income backgrounds. Access to novel agents is similarly inequitable, influenced by insurance status, geographic location, and systemic healthcare barriers. Addressing these disparities is essential to ensure equitable improvements in outcomes [9].

The management of CLL is increasingly complex, requiring individualized therapeutic strategies that account for patient comorbidities, toxicity profiles, and evolving treatment landscapes. Efforts to refine therapeutic sequencing and broaden access to novel therapies will be critical in optimizing care for all patients with CLL [77].

## 7. Future Strategies Must Prioritize

Future strategies must prioritize biologically informed, adaptive treatment approaches to further improve the durability of response and overcome therapeutic resistance in CLL.

MRD-guided therapy represents a critical step toward individualized treatment duration. Incorporating sensitive MRD assessment into clinical decision-making allows for treatment discontinuation based on depth of response rather than arbitrary fixed timelines. This approach may maximize long-term disease control while minimizing cumulative toxicity, treatment fatigue, and cost, particularly in combination regimens capable of achieving deep MRD negativity [78].

Expanded genomic profiling is essential to anticipate resistance and guide treatment selection. Baseline and longitudinal assessment of BTK, PLCγ2, TP53, complex karyotype, and emerging resistance-associated mutations (e.g., XPO1) can enable early identification of high-risk patients and inform timely therapeutic transitions before clinical progression occurs. Integration of serial genomic monitoring into routine practice may help prevent clonal evolution under therapeutic pressure [79,80].

Inclusion of diverse and underrepresented populations in clinical trials remains a major unmet need. Many pivotal CLL studies underrepresent racial and ethnic minorities, older adults, and patients with comorbidities, limiting the generalizability of trial outcomes. Broadening trial eligibility and expanding community-based enrollment are critical to ensuring equitable access to novel therapies and improving outcomes across hereditary risk cancer populations [9].

Triplet therapies and next-generation BTKis offer promising strategies to overcome resistance. Rational combination approaches targeting complementary survival pathways may deepen responses and suppress resistant subclones, while next-generation BTKis retain activity against C481 and non-C481 mutations. These advances, along with emerging BTK degraders, are likely to redefine sequencing strategies and improve outcomes in patients with relapsed or high-risk disease [32].

## 8. Conclusions

CLL management has entered an era dominated by targeted, chemotherapy-free therapies, yet optimal treatment sequencing remains complex. Precision-based strategies incorporating genomic profiling, MRD-guided decision-making, and resistance-aware sequencing are increasingly central to durable disease control. Time-limited combinations, triplet regimens, and next-generation BTKis capable of overcoming acquired resistance represent key advances poised to reshape clinical practice. Future studies must focus on individualized treatment strategies based on MRD status and molecular risk, aiming to balance efficacy, safety, and quality of life.

## Data Availability

The original contributions presented in this study are included in the article. Further inquiries can be directed to the corresponding author.
